# Predicting Hazardous Alcohol Drinking Behaviors in Family Members of Hazardous Alcohol-Drinker Patients

**DOI:** 10.3390/ijerph19095497

**Published:** 2022-05-01

**Authors:** Ching-Yen Chen, Chen-Chun Lin, Jung-Ta Kao, Wen-Ling Yeh, Chiao-Yun Lin, Yun-Fang Tsai

**Affiliations:** 1 Department of Psychiatry, Chang Gung Memorial Hospital at Keelung, Keelung City 204, Taiwan; psycychen@adm.cgmh.org.tw; 2 College of Medicine, Chang Gung University, Taoyuan City 333, Taiwan; lincc53@adm.cgmh.org.tw (C.-C.L.); yeh610128@gmail.com (W.-L.Y.); 3 Department of Hepato-Gastroenterology, Chang Gung Memorial Hospital at Linkou, Taoyuan City 333, Taiwan; 4 School of Medicine, College of Medicine, China Medical University, Taichung City 404, Taiwan; garrydarkao@gmail.com; 5 Division of Hepato-Gastroenterology, Department of Internal Medicine, China Medical University Hospital, Taichung City 404, Taiwan; 6 Department of Orthopedics, Lotung Poh-Ai Hospital, Luodong Township, Yilan County 265, Taiwan; 7 Department of Nursing, Chang Gung Memorial Hospital at Linkou, Taoyuan City 333, Taiwan; michera@cgmh.org.tw; 8 School of Nursing, College of Medicine, Chang Gung University, 259, Wen-Hwa 1st Road, Taoyuan City 333, Taiwan; 9 Department of Nursing, Chang Gung University of Science and Technology, Taoyuan City 333, Taiwan

**Keywords:** alcohol, hazardous drinking behaviors, family member, protective factor

## Abstract

Family members of hazardous or harmful alcohol drinkers suffer many consequences of their relative’s alcohol-drinking behaviors and risk developing their own hazardous alcohol drinking behaviors. Studies of alcohol-related healthcare problems have mainly focused on patients, with few studies on their family members. This cross-sectional study explored factors predicting hazardous alcohol drinking behaviors in family members of hazardous alcohol-drinker patients. Participants were recruited from four randomly chosen hospitals in Taiwan. Data were collected using self-report questionnaires on family members’ alcohol use, perceived stress, coping mechanisms, social support, health, quality of life, protective factors against hazardous alcohol drinking, facilitative factors for hazardous alcohol drinking, and demographics. The 318 family members who participated in this study were divided by their Chinese-version Alcohol Use Disorders Identification Test scores into two groups: hazardous alcohol drinkers (score ≥ 8) and non-hazardous alcohol drinkers (score < 8). Significant factors predicting hazardous alcohol drinking behaviors were found by logistic regression to be the frequency of using general coping mechanisms (OR = 1.29, *p* < 0.01), the frequency of using strategies to cope with patients’ drinking-related behaviors (OR = 0.89, *p* < 0.01), factors protecting against hazardous alcohol drinking (OR = 0.76, *p* < 0.01) and factors facilitating hazardous alcohol drinking (OR = 1.52, *p* < 0.01). Interventions should be designed for family members of hazardous alcohol drinkers to address these four significant predictors.

## 1. Introduction

Drinking alcohol to excess severely affects individuals and society [1]. For example, family members of hazardous or harmful alcohol drinkers suffer negative effects, including a greater risk of developing alcoholism [2], adverse behavioral and health outcomes [3], stress-related physical and psychological symptoms [4,5], and greater medical costs [6]. Moreover, romantic partners, defined as those cohabiting, engaged, or married, positively influenced each other’s heavy episodic alcohol drinking over a three-year follow-up [7]. Thus, family members of identified hazardous or harmful alcohol drinker patients should be screened and targeted for treatment [8]. Hazardous and harmful alcohol drinkers, so-called because their alcohol drinking harms their health, are identified by scores ≥ 8 on the Alcohol Use Disorders Identification Test (AUDIT) [9].

Studies of alcohol-related healthcare problems in Western countries have mainly focused on patients, with few studies on their family members [4,10]. These studies involved four types of interventions targeted at family members of a relative affected by the use of alcohol: (1) working with affected family members to increase the substance user’s engagement in treatment [11], (2) engaging both users and family members in treatment to reduce users’ substance abuse [12,13], (3) focusing on the needs of affected family members to reduce their physical and psychological symptoms [4], and (4) involving family members in enhancing the substance user’s engagement in treatment, reducing their alcohol use, and improving family members’ mood and functions [14]. None of these interventions focused on family members’ drinking behaviors.

An appropriate setting for detecting hazardous or harmful alcohol drinkers is general hospitals [15]. Indeed, hazardous alcohol drinker patients have been detected in general hospitals in Western studies at 12–26% prevalence [15,16]. However, these studies do not report the prevalence of hazardous alcohol drinking for family members of these patients. In Taiwan, the prevalence of patients with hazardous alcohol drinking reported for general hospitals ranges from 5.7–19.2%, depending on the hospital units surveyed [17,18,19]. We found only one study that explored risk factors for hazardous alcohol drinking in family members of Chinese patients who were hazardous alcohol drinkers [20]. These family members were found to have a 13.3% prevalence of hazardous alcohol drinking problems, with five risk factors identified: male gender, low education level, heart disease, smoking, and chewing betel quid [20]. Only two behavioral (modifiable) risk factors were identified: smoking and chewing betel quid.

However, interventions targeting modifiable risk factors are necessary to effectively reduce hazardous alcohol-drinking behaviors in family members of hazardous alcohol drinker patients. Cognitive theory, which is rooted in depression, has been suggested for application in groups of patients diagnosed with various conditions including hazardous alcohol drinking behaviors [21]. This theory emphasizes that one’s beliefs and expectancies are related to whether one achieves the goal of a specific behavior [21]. On the one hand, the beliefs and expectations of hazardous alcohol drinkers about hazardous alcohol-drinking behaviors can be considered facilitative factors enhancing hazardous alcohol-drinking behaviors. On the other hand, the beliefs and expectations of non-hazardous alcohol drinkers about hazardous alcohol-drinking behaviors can be considered protective factors against hazardous alcohol-drinking behaviors.

Thus far, no model for hazardous alcohol drinking behaviors among family members of patients who are hazardous alcohol drinkers is available either in Western countries or in Taiwan. Such a model could help healthcare providers understand the factors influencing hazardous alcohol drinking behaviors in these family members to design suitable interventions to prevent them from hazardous alcohol drinking. Filling this gap is an urgent task. One model often used in Western countries to understand the needs of family members of alcohol drinker patients and reduce their physical and psychological symptoms is the stress-strain-coping-support model [10,22,23]. This model assumes that an alcohol problem in the family creates chronic stress for family members, which they try to cope with by seeking support from professional and informal resources [22,23]. However, this model has been mainly applied to family members of patients addicted to drugs and alcohol, focused on substance abuse-related stress and coping, and was not linked to family members’ drinking behaviors. Here, we argue that general stress and coping behaviors should also be considered for family members of patients with hazardous alcohol drinking behaviors.

We hypothesized that family members’ perceived stress (in general and specifically related to hazardous alcohol-drinker patients), coping (in general and specifically related to hazardous alcohol-drinker patients), social support (professional and informal), health condition (physical and psychological symptoms, quality of life), and beliefs about hazardous alcohol drinking (protective factors against hazardous alcohol drinking and facilitative factors for hazardous alcohol drinking) each play a role in their drinking behaviors. Therefore, the purpose of this study was to examine the degree of these factors in predicting family members’ hazardous alcohol-drinking behaviors.

## 2. Methods

### 2.1. Design

This cross-sectional study, which was part of a large series of studies on family members of hazardous alcohol-drinker patients in Taiwan, is reported according to the guidelines of Strengthening the Reporting of Observational Studies in Epidemiology (STROBE) [24].

### 2.2. Sample and Setting

Four hospitals in northern Taiwan were randomly chosen. A sample of hazardous alcohol-drinking patients was from referrals by physicians or nurses from psychiatric, gastrointestinal medical-surgical, trauma, and rehabilitation clinics and wards where most patients with alcohol problems are seen in Taiwan [25]. Patients were included using these criteria: (1) Chinese-version AUDIT score ≥ 8 [25], (2) >20 years old, and (3) able to speak Chinese/Taiwanese. Patients who met the hazardous alcohol-drinking criteria were referred to a research assistant (RA) if they were interested in referring family members for our study. Family members were included if they met these criteria: (1) >20 years old, (2) parent, sibling, child, or partner of a hazardous alcohol drinker patient, and (3) able to speak Chinese/Taiwanese. Statistical power for logistic regression requires a sample size of at least 10 participants for each predictor [26]. In the present study, we examined 19 predictors (demographic, n = 10 and related factors, n = 9) and estimated that at least 190 family members would need to be recruited.

### 2.3. Ethical Approval

The study was approved by the institutional review boards (IRBs) of Chang Gung Medical Foundation (102-5414B) and the China Medical University Hospital (CMUH103-REC3-094) prior to initiating this study.

### 2.4. Measures

#### 2.4.1. Assessment of Alcohol Use

Alcohol use for family members of hazardous alcohol drinker patients was measured by the Chinese version of the AUDIT questionnaire [25]. The 10-item AUDIT is a screening tool that assesses alcohol-related behaviors in the previous year: alcohol consumption (3 items, such as, “How often do you have a drink containing alcohol?”); drinking behaviors (3 items, such as, “How often during the last year have you found that you were not able to stop drinking once you had started?”); and alcohol-related problems (4 items, such as, “Have you or somebody else been injured as a result of your drinking?”). Responses are rated on a 5-point Likert scale from 0 to 4, for a maximum score of 40 points. Higher AUDIT scores indicate a more severe level of risk; scores ≥ 8 indicate a tendency to hazardous or harmful alcohol use. The reliability of the Chinese version was established in 112 hospitalized patients in Taiwan [25].

#### 2.4.2. Assessment of Perceived General Stress

The degree to which participants appraise situations in their life as stressful was measured by the Chinese version of the Perceived Stress Scale (PSS) [27]. The PSS has been widely used in various patients including those with colorectal cancer [28], coronary heart disease [29], and the general population. Each of the 14-items of the PSS is a statement, such as, “How often have you been upset because of something that happened unexpectedly?” Items are scored on a 5-point Likert scale from 0 to 4; scores range from 0 to 56. Higher PSS scores indicate more severe perceived stress. Cronbach’s α coefficient for the total scale was 0.85. The test-retest reliability was 0.85 [27].

#### 2.4.3. Assessment of Perceived Stress from the Hazardous-Drinker Patient

The degree to which participants appraise stress from their hazardous-drinker family member was measured by the Perceived Stress Questionnaire for Family Members of a Hazardous-Drinker Patient, which was developed by the research team. This 14-item questionnaire is comprised of statements of alcohol-related concerns about the hazardous-drinker patient that cause stress using four subscales: worry about the patient (5 items, such as, “I worry about his/her health condition”); unease about the current and future situation (3 items, such as, “I Don’t dare to let relatives and friends know about the problems he/she has caused”); impact on children (3 items, such as “I worry about our children being beaten and scolded”); and impact on finances (3 items, such as “I have been forced to take on the responsibility of supporting the family”). Each item is rated from 0 (no stress) to 3 (severe stress). Perceived stress scores are calculated by adding the total score for the 14 item scores; higher scores indicate more perceived stress. Cronbach’s α coefficients for the total scale and its four subscales were 0.81, 0.78, 0.77, 0.79, and 0.94, respectively. The intraclass correlation coefficients (ICCs) of the total scale and its four subscales over 4 weeks were 0.91, 0.91, 0.89, 0.96, and 0.67, respectively.

#### 2.4.4. Assessment of General Coping Mechanisms

Family members’ general coping behaviors were measured by the Coping Strategy Questionnaire (CSQ) [30], which was originally used in patients with chronic obstructive pulmonary disease and modified by the research team. The 40-item CSQ has two dimensions: (1) problem-based approach (15 items, such as “Discuss the problem with other family members or friends”) and (2) emotion-based approach (25 items, such as “Blame others”). Response options are rated on a 4-point Likert scale (0–3), for a maximum score of 120 points. Higher CSQ scores indicate more frequently used coping strategies. Cronbach’s α coefficients for the total scale and its two subscales were 0.82, 0.83, and 0.85, respectively.

#### 2.4.5. Assessment of Strategies for Coping with Patients’ Drinking-Related Behavior

Family members’ strategies for coping with patients’ drinking behaviors were measured by the Coping Questionnaire for Family Members of a Hazardous-Drinker Patient, which was developed by the research team. Each item of this 24-item scale is a statement about how the participant copes with drinking behaviors, which is comprised of five subscales: maintaining good interactions (7 items, such as I try to be nice to him/her and hope he/she will change their mind); seeking help (6 items, such as, “I seek support and assistance from family or friends”); shifting attention (5 items, such as, “I shift my attention by engaging in work or household chores”); taking it one day at a time (3 items, such as, “I tell myself to let it [the behavior] go; don’t be so serious”); and avoiding contact and alienation (3 items, such as, “I try to reduce my emotional connections”). Each item is rated from 0 (never used) to 3 (most frequently used). Coping-strategy scores are calculated by adding the 24 item scores; higher scores indicate using more coping strategies. Cronbach’s α coefficients of the total scale and its five subscales were 0.80, 0.78, 0.78, 0.69, 0.79, and 0.71, respectively. The ICCs of the total scale and its five subscales over 4 weeks were 0.90, 0.92, 0.91, 0.87, 0.88, and 0.85, respectively.

#### 2.4.6. Assessment of Social Support

Family members’ social support was measured by the Social Support Scale (SSS) [31], which was originally used in heart transplant recipients and modified by the research team. The SSS has 16 items that measure two social support resources (professional and informal) and four patterns of support (emotional, 4 items, such as “They comfort me when I feel the need”; informational, 4 items, such as “They provide information about caring for patients”; appraisal, 4 items, such as “They respect my decision”; and material, 4 items, such as “They share my work when I’m physically or mentally unwell”). Total scores range from 0–48, with higher scores representing better social support. Cronbach’s α coefficients for the total scale and its two subscales were 0.85, 0.82, and 0.84, respectively.

#### 2.4.7. Assessment of Mental Health

The mental health of family members was assessed using the Chinese Health Questionnaire (CHQ) [32]. The 30-item CHQ has four dimensions: (1) anxiety and worrying (e.g., Been taking things hard?), (2) somatic symptoms (e.g., Had discomfort or a feeling of pressure in your chest?), (3) social dysfunction (e.g., Felt in general things are well managed?), and (4) depression (e.g., Been feeling that life is entirely hopeless?). Response options are rated on a 4-point Likert scale. Total scores range from 0–30, with higher scores representing worse mental health. The reliability and validity of the CHQ are well established [32].

#### 2.4.8. Assessment of Quality of Life

Family members’ quality of life was measured by the SF-36 Taiwan version [33]. The SF-36 is considered one of the most well-established generic health-related quality-of-life measures. The SF-36 has 36 items that measure patients’ physical functioning (e.g., Does your health limit you in these activities?), social functioning (e.g., To what extent has your physical health or emotional problems interfered with your normal social activities with family, friends, neighbors, or groups?), role limitations due to physical problems (e.g., Have you had any of the following problems with your work or other regular daily activities as a result of your physical health?), role limitations due to emotional problems (e.g., have you had any of the following problems with your work or other regular daily activities as a result of any emotional problems?), mental health (e.g., Have you been a very nervous person?), vitality (e.g., Did you feel full of pep?), pain (e.g., How much bodily pain have you had), and general health perceptions (e.g., In general, would you say your health is). The reliability and validity of the SF-36 are well established [33].

#### 2.4.9. Assessment of Protective Factors against Hazardous Alcohol Drinking

Participants’ protective factors against hazardous alcohol drinking were measured by the Family Members’ Protective Factors against Hazardous Alcohol Drinking Questionnaire, which was developed by the research team. This 18-item questionnaire has five subscales: perceptions of alcohol (3 items, such as “Drinking alcohol is bad for your health”), impact on family (4 items, such as “People who consume alcohol are bad role models for children”), perceptions of stress and coping (5 items, such as “I don’t have stress, and have no need to drink”), impact on health and work (3 items, such as “Alcohol has an impact on your work”), and impact on relationships with others (3 items, such as “Alcohol can cause conflicts or disputes among friends”). Each item is rated from 1 (highly disagree) to 5 (highly agree). Protective-factor scores are calculated by adding the 18-item scores; higher scores indicate more protective factors. Cronbach’s α coefficients for the total scale and its five subscales were 0.90, 0.86, 0.75, 0.82, 0.76, and 0.77, respectively. The ICCs for the total scale and its five subscales over 4 weeks were 0.94, 0.94, 0.93, 0.91, 0.90, and 0.91, respectively.

#### 2.4.10. Assessment of Facilitative Factors for Hazardous Alcohol Drinking

Participants’ facilitative factors for hazardous alcohol drinking were measured using the Family Members’ Facilitative Factors for Hazardous Alcohol Drinking Questionnaire, which was developed by the research team. This 13-item questionnaire has three subscales: relieving stress (4 items, such as “It helps to relax”), social interactions (5 items, such as “It’s a social activity”), and personal and family activities (4 items, such as “It’s the way the family gets together”). Each item is rated from 1 (highly disagree) to 5 (highly agree). Facilitative factors scores are calculated by adding the 13 item scores; higher scores indicate more facilitative factors. Cronbach’s α coefficients for the total scale and its three subscales were 0.82, 0.81, 0.79, and 0.72, respectively. The ICCs of the total scale and three subscales over 4 weeks were 0.95, 0.93, 0.95, and 0.90, respectively.

#### 2.4.11. Assessment of Demographic Information

Data on family members’ age, gender, education level, marital status, living status, relationship with the hazardous-drinker patient, smoking and betel quid-chewing behaviors, several chronic diseases, and drinking patterns were collected using a demographic information form [8].

### 2.5. Procedure

The institutional review board of each hospital individually approved the study, after which physicians or nurses in the study settings referred hazardous alcohol drinker patients. Referred cases were approached by a RA who described the study, screened them for inclusion criteria, obtained their written consent to participate, and asked them to complete the demographic form as well as to refer family members for study participation. Then, the RA approached family members, described the study, screened them for inclusion criteria, and obtained written consent to participate. The RA distributed and collected questionnaires from the family members who provided written informed consent.

### 2.6. Data Analysis

Data were analyzed using SPSS for Windows, version 23.0 (SPSS, Chicago, IL, USA). Descriptive statistics were performed to identify the demographic characteristics of participants. The chi-square tests and *t*-tests were performed to examine the group differences (family members without and with hazardous alcohol-drinking behaviors) in demographics and related factors. Finally, variables that differed significantly in the univariate analysis were selected as independent variables. Logistic regression was performed to identify the predictors of family members’ hazardous drinking behaviors.

## 3. Results

### 3.1. Family Members’ Characteristics

Of 333 patients approached who were hazardous alcohol drinkers, 321 agreed to refer family members to participate; 12 patients refused to refer family members due to time constraints. Patients referred 325 family members; four declined to participate due to time constraints, and three could not be reached. Finally, 318 family members of hazardous-drinker patients participated in this study. Family members were divided into two groups, based on their scores on the Chinese version of the AUDIT: hazardous alcohol drinkers (AUDIT score ≥ 8) and non-hazardous alcohol drinkers (AUDIT score < 8). Most non-hazardous alcohol drinkers were female (n = 228, 87.11%); most hazardous alcohol drinkers were male (n = 39, 62.90%). Both groups differed significantly by age, gender, educational level, religious beliefs, whether they lived with the patient, their relationship to the patient, smoking, chewing betel quid, and the number of chronic diseases (all *p* ≤ 0.01) (Table 1).

### 3.2. Related Factors

Data from survey questionnaires of related factors for family member groups having non-hazardous and hazardous drinking behaviors are shown in Table 2. Family members of hazardous drinker patients had a moderate level of perceived general stress, which did not differ significantly between groups. However, perceived stress from the hazardous-drinker patient was significantly lower for participants with hazardous drinking behaviors compared with the non-hazardous behavior group (*p* < 0.01). Mean scores for frequency of using general coping strategies were significantly higher for the hazardous drinking behaviors group (*p* < 0.01), whereas for frequency of using coping strategies to cope with the patient’s drinking-related behavior was significantly lower for the group with hazardous drinking behaviors compared with the non-hazardous group (*p* < 0.01). This low level of coping with drinking-related behaviors is reflected in the frequency of all participants who responded, “most frequently”, to the statements, “I adjust to my own lifestyle” (95/318, 29.9%), and “I wait until he/she awakes from being drunk, then we communicate with each other” (91/318, 28.6%). All participants had moderate levels of informal social support, mental health, and acceptable quality of life (overall and physical), and mean scores did not differ between groups. However, mean scores were higher for professional social support and lower for psychological quality of life compared with non-hazardous drinkers (both *p* < 0.05). Mean total scores were significantly lower for the hazardous drinking group on the Protective Factors against Hazardous Alcohol-Drinking Questionnaire (57.63, SD = 5.80) compared with the non-hazardous group (70.73, SD = 7.32, *p* < 0.01). The highest mean scores were for the subscale related alcohol use (M = 4.10, SD = 0.56), followed by the impact on family (M = 3.90, SD = 0.51). Mean total scores were significantly higher for the hazardous drinking group on the Family Members’ Facilitative Factors for Hazardous Alcohol Drinking Questionnaire compared with the non-hazardous group (*p* < 0.01). For all participants the highest mean subscale scores were for social interactions (M = 2.96, SD = 0.53), followed by personal and family activities (M = 2.86, SD = 0.45).

### 3.3. Predictors of Family Members’ Hazardous Alcohol Drinking Behaviors

Variables that differed significantly in univariate analysis between non-hazardous and hazardous alcohol drinkers were selected as independent variables for logistic regression, which included demographic variables (age, gender, educational level, religious beliefs, whether they lived with the patient, their relationship to the patient, smoking, chewing betel quid, and the number of chronic diseases) as well as perceived stress from the hazardous-drinker patient, frequency of using general coping strategies, frequency of using strategies to cope with patient’s drinking-related behaviors, professional social support, psychological aspect for quality of life, protective factors against hazardous alcohol-drinking behaviors, and facilitating factors enhancing hazardous alcohol-drinking behaviors. Analysis indicated significant factors predicting hazardous alcohol-drinking behaviors were the frequency of using general coping mechanisms (OR = 1.29, *p* < 0.01), the frequency of using strategies to cope with patients’ drinking-related behaviors (OR = 0.89, *p* < 0.01), factors protecting against hazardous alcohol drinking (OR = 0.76, *p* < 0.01), and factors facilitating hazardous alcohol drinking (OR = 1.52, *p* < 0.01) (Table 3).

## 4. Discussion

Our results revealed that family members’ hazardous alcohol-drinking behaviors were significantly predicted by the frequency of using general coping mechanisms, the frequency of using strategies to cope with patients’ hazardous alcohol drinking-related behaviors, factors protecting against hazardous alcohol drinking, and factors facilitating hazardous alcohol drinking. These findings support the stress-strain-coping-support model of how stress is mediated by coping in family members affected by another family member’s addictive behavior [10,22,23] and contribute to developing interventions for family members’ hazardous alcohol-drinking behaviors.

Univariate analysis showed family members without and with hazardous alcohol-drinking behaviors differed significantly for several demographic variables measured. While the difference in mean age between groups may have no clinical significance, the difference in gender between groups is an important issue. The prevalence rate of hazardous alcohol-drinking behaviors was higher for males than females in our study, which reflects findings of a review of Western studies [15] as well as a study of Taiwanese family members of patients with hazardous alcohol-drinking [20]. However, studies have suggested the consequences of heavy alcohol use, or alcohol use disorders, appear to have more negative repercussions for women than men [34]. Therefore, gender should be considered when designing alcohol interventions.

In our study, a higher educational level was also associated with hazardous alcohol-drinking behaviors, which is contradictory to previous studies [20,35]. Those who were Taoists in this study were also more likely to have hazardous alcohol-drinking behaviors. Hazardous alcohol use has been associated with having no religious beliefs in Western studies [36], therefore it is unclear why Taoists had higher hazardous alcohol-drinking behaviors. Further studies are needed to explore the role of education level and religious beliefs in family members’ hazardous alcohol-drinking behaviors.

Smoking tobacco and betel quid use were associated with hazardous alcohol drinking among our participants. An increasing prevalence of smoking has been associated with an increasing level of alcohol consumption [37]. Chewing betel quid has a thousand-year history in Taiwan and is mainly a habit of blue-collar workers [38]. Betel quid chewing has also been shown to synergistically enhance the carcinogenic effects of smoking [39]. Taken together, smoking, betel quid chewing, and drinking alcohol are deleterious to oral health [40]. These participants may be vulnerable to developing oral cancer and other chronic diseases [37].

When univariate analysis examined factors related to non-hazardous and hazardous alcohol drinking behaviors, family members without hazardous alcohol drinking behaviors perceived higher stress from the patient who was a hazardous alcohol drinker but had less social support from professionals. Although their psychological aspect of quality of life was better than family members with hazardous alcohol drinking behaviors, healthcare providers should be aware of this deficit and provide sufficient social support for this population.

Our results show that the frequency of using general coping mechanisms was associated with an increase in family members’ hazardous alcohol-drinking behaviors, but the frequency of using strategies to cope with the patient’s drinking-related behavior was associated with a decrease in their hazardous alcohol-drinking behaviors. These results suggest that using only general coping mechanisms are not enough to cope with family members’ stress from patients’ drinking-related behaviors. These family members need help developing strategies to cope with patients’ drinking-related behavior. Our sample of family members was also inclined to use various strategies to cope with the patients’ hazardous-drinking behaviors. Future studies may examine the effects of different family members’ strategies to cope with patients’ hazardous alcohol-drinking behaviors.

This study also found that factors protecting against hazardous alcohol drinking were associated with decreasing hazardous alcohol-drinking behaviors, but factors facilitating hazardous alcohol drinking were associated with increasing hazardous alcohol drinking behaviors. The ideas of protective and facilitative factors are rooted in depression and have been suggested for application in other patient groups, e.g., those with anxiety [21]. Our study results can also be applied to predicting the behaviors of family members who become hazardous alcohol drinkers. Protective behavioral strategies are commonly used by college students to reduce their heavy drinking and alcohol-related consequences [41]. Unlike college students, family members of hazardous alcohol drinkers live longer with their hazardous alcohol-drinking relatives and experience their alcohol-drinking-related behaviors over a longer period. Their coping strategies are likely to be different.

Examination of the Family Members’ Protective Factors against Hazardous Alcohol-Drinking Questionnaire shows that our family members tended to have a negative impression of drinking alcohol and worried about the negative impact of alcohol drinking on their family members, including on their health and behaviors. In contrast, examining of the Family Members’ Facilitative Factors for Hazardous Alcohol Drinking Questionnaire, maintaining social interactions as well as personal and family activities were the main facilitative factors for their hazardous alcohol-drinking behaviors. Future studies should consider enhancing family members’ protective factors and decreasing their facilitative factors to decrease their hazardous alcohol-drinking behaviors.

Other factors that were significantly different in the univariate analysis, but not included in the final regression model consisted of age, gender, educational level, religious beliefs, whether they lived with the patient, their relationship to the patient, smoking, chewing betel quid, and the number of chronic diseases, their perceived stress from the hazardous-drinker patient, professional social support, and psychological quality of life. This population has not been widely studied; therefore, we can only compare our findings to one previous Taiwanese study which identified five risk factors for hazardous drinking: male gender, low education level, heart disease, smoking, and chewing betel quid [20]. Based on the literature review, cognitive theory, and the stress-strain-coping-support model, we chose the most related factors and used 10 questionnaires as measurement instruments. However, these were already very long and not suitable for adding more items. Since study variables differed, it may cause different results. Future studies could explore the relationships between personal factors of family members of hazardous alcohol drinkers and their own hazardous alcohol-drinking behaviors. However, our original Coping Questionnaire for Family Members of a Hazardous-Drinker Patient included one item, “seeking help from health care providers,” which was the sample’s second-highest commonly used strategy (96/318, 30.2%). This item was deleted from the final questionnaire due to a validity test (factor analysis). However, such an item was recently supported by the finding that New Zealand family members affected by a relative’s substance misuse were found to cope with this misuse by seeking timely access to evidence-based information, enhancing personal coping strategies, and accessing informal and formal support [42]. These findings and ours highlight the importance of healthcare providers offering timely information and support for family members of hazardous alcohol drinkers.

### Limitations

Although this study provides important information about predictors of hazardous alcohol-drinking behaviors in family members of hazardous alcohol-drinker patients, it was limited by the sample being recruited by convenience from four randomly selected hospitals in Taiwan. Future studies should use random sampling. In addition, several questionnaires were developed by the research team. Although total and subscale scores had an acceptable Cronbach’s α coefficient, further studies should be conducted to establish the validity of the research-developed surveys.

## 5. Conclusions

Results of this study revealed that the hazardous alcohol-drinking behaviors of family members of hazardous alcohol-drinker patients were significantly predicted by the frequency of using general coping mechanisms, the frequency of using strategies to cope with patients’ drinking-related behaviors, factors protecting against hazardous alcohol-drinking, and factors facilitating hazardous alcohol-drinking. Interventions are needed to help family members of hazardous alcohol drinkers develop strategies to cope with patients’ drinking-related behaviors, enhance their protective factors against hazardous alcohol drinking and decrease their facilitative factors to decrease their own hazardous alcohol-drinking behaviors.

## Figures and Tables

**Table 1:** Demographics of family members without and with hazardous alcohol-drinking behaviors (N = 318).

	Non-Hazardous Alcohol Drinkers	Hazardous Alcohol Drinkers	
Variable	(n = 256)	(n = 62)	t/χ^2^ (*p*)
Age, years (mean, SD)	49.69 (13.13)	45.03 (12.03)	2.55 (0.01) ^a^
Gender (n, %)			71.28 (<0.01) ^b^
Male	33 (12.89)	39 (62.90)	
Female	223 (87.11)	23 (37.10)	
Education level (n, %)			32.00 (<0.01) ^b^
≤Primary school	65 (25.39)	5 (8.06)	
Junior high school	42 (16.41)	4 (6.45)	
Senior high school	70 (27.34)	10 (16.13)	
≥College or above	79 (30.86)	43 (69.35)	
Marital status (n, %)			1.86 (0.40) ^b^
Single	39 (15.23)	11 (17.74)	
Married	206 (80.47)	46 (74.19)	
Other	11 (4.30)	5 (8.06)	
Religious belief (n, %)			48.32 (<0.01) ^b^
Buddhism	35 (13.67)	9 (14.52)	
Taoism	37 (14.45)	30 (48.39)	
Belief in God but no specific religion	165 (64.45)	13 (20.97)	
Other	19 (7.42)	10 (16.13)	
Living with patient (n, %)			31.12 (<0.01) ^b^
Yes	244 (95.31)	45 (72.58)	
No	12 (4.69)	17 (27.42)	
Relation to the patient (n, %)			20.07 (<0.01) ^b^
Parent	23 (8.98)	4 (6.45)	
Sibling	13 (5.08)	12 (19.35)	
Child/Daughter-in-law	47 (18.36)	18 (29.03)	
Partner	173 (67.58)	28 (45.16)	
Smoking (n, %)			48.76 (<0.01) ^b^
Yes	29 (11.33)	31 (50.00)	
No	227 (88.67)	31 (50.00)	
Chewing betel quid (n, %)			7.62 (0.01) ^b^
Yes	231 (90.23)	48 (77.42)	
No	25 (9.77)	14 (22.58)	
Chronic diseases (n, %)	0.45 (0.81)	0.81 (0.99)	−2.97 (<0.01) ^a^

Note: SD = standard deviation; betel quid: it generally contains betel leaf, areca nut, and slaked lime. ^a^ *t*-test; ^b^ Chi-square test.

**Table 2:** Factors related to family members without and with hazardous alcohol-drinking behaviors (N = 318).

	Non-Hazardous Alcohol Drinkers (n = 256)	Hazardous Alcohol Drinkers (n = 62)	
Variable	Mean (SD)	Mean (SD)	t (*p*)
Perceived general stress	30.40 (3.12)	30.37 (2.54)	0.06 (0.95)
Perceived stress from hazardous-drinker patient	16.20 (9.79)	5.27 (8.07)	8.14 (<0.01)
Frequency of using general coping strategies	43.83 (4.03)	45.61 (3.80)	−3.17 (<0.01)
Frequency of using strategies to cope with patient’s drinking-related behavior	18.79 (10.10)	4.89 (7.26)	10.21 (<0.01)
Social support-informal	20.78 (4.46)	20.10 (4.49)	1.08 (0.28)
Social support- professional	24.69 (4.50)	26.21 (4.76)	−2.36 (0.02)
Mental health	17.36 (4.00)	18.45 (4.50)	−1.88 (0.06)
Quality of life	82.97 (6.09)	82.08 (6.21)	1.03 (0.31)
Physical aspect	46.27 (4.49)	46.70 (4.57)	−0.68 (0.50)
Psychological aspect	36.70 (3.51)	35.38 (3.27)	2.69 (0.01)
Factors protecting against hazardous alcohol drinking	70.73 (7.32)	57.63 (5.80)	13.13 (<0.01)
Factors facilitating hazardous alcohol drinking	35.94 (4.75)	43.63 (4.52)	−11.55 (<0.01)

Note: SD = standard deviation.

**Table 3:** Predictors of hazardous alcohol drinking behaviors among family members (N = 318).

						95% CI for OR	
Variable	B	SE	Wald	*p*	OR	Lower	Upper
Frequency of using general coping strategies	0.25	0.07	12.60	<0.01	1.29	1.12	1.48
Frequency of using strategies to cope with patient’s drinking-related behavior	−0.12	0.04	9.88	<0.01	0.89	0.82	0.96
Factors protecting against hazardous alcohol drinking	−0.28	0.05	28.60	<0.01	0.76	0.69	0.84
Factors facilitating hazardous alcohol drinking	0.42	0.09	21.30	<0.01	1.52	1.27	1.81

Note: OR = odds ratio; CI = confidence interval.

## Data Availability

The data presented in this study are available on request due to privacy restrictions.
